# Upregulation of miR-214 Induced Radioresistance of Osteosarcoma by Targeting PHLDA2 via PI3K/Akt Signaling

**DOI:** 10.3389/fonc.2019.00298

**Published:** 2019-04-18

**Authors:** Yi Li, Xinmao Song, Zegang Liu, Qiutian Li, Meijin Huang, Bin Su, Yuchi Mao, Yuanyuan Wang, Wenqian Mo, Hong Chen

**Affiliations:** ^1^Department of Oncology, 920th Hospital of Joint Logistics Support Force, Kunming, China; ^2^Department of Radiation Oncology, Eye, Ear, Nose, and Throat Hospital, FuDan University, Shanghai, China; ^3^Department of General Surgery, 920th Hospital of Joint Logistics Support Force, Kunming, China; ^4^Department of Pathology, 920th Hospital of Joint Logistics Support Force, Kunming, China

**Keywords:** miR-214, osteosarcoma, PHLDA2, PI3K/Akt pathway, radiosensitivity

## Abstract

Osteosarcoma is an aggressive bone tumor with high resistance to radiotherapy. Pleckstrin homology-like domain family A member 2 (PHLDA2) displays low expression in human osteosarcoma as a proapoptosis factor. miRNAs have been shown to be important in modulating translation and therapeutic responsiveness in solid tumors. Herein, we used luciferase assay to show that miR-214 downregulates the PHLDA2 expression by targeting its 3′-untranslated region (UTR). A high level of miR-214 was identified in tumor tissues from 30 osteosarcoma patients via qPCR analysis, associated positively with lung metastasis. Ectopic expression miR-214 enhanced radioresistance in osteosarcoma cells, with decreased IR-induced apoptosis. Moreover, the depletion of miR-214 enhanced radiosensitivity in both osteosarcoma cells and mouse xenograft models. Importantly, we showed that miR-214 regulated the activation of phosphatidylinositol-3-kinase/Akt signaling pathway by inhibiting PHLDA2. Finally, the introduction of PHLDA2 cDNA lacking the 3′-UTR or treatment with Akt inhibitor LY294002 partially abrogated miR-214-induced radioresistance. In summary, our results reveal that the upregulation of miR-214 as a frequent event in osteosarcoma contributes to radioresistance by regulating the PHLDA2/Akt pathway. The miR-214/PHLDA2/Akt axis provides a new avenue toward understanding the mechanism of radiosensitivity and may be a potential target for osteosarcoma intervention.

## Introduction

Osteosarcoma (OS) is the most common primary bone tumor in children and adolescents. Neoadjuvant chemotherapy, followed by surgical resection and additional adjuvant chemotherapy, is the typical treatment approach for resectable high-grade osteosarcoma. Radiotherapy could be an important option as a local treatment of unresectable tumors, before surgery or with aggressive chemotherapy to reduce recurrence (1). However, resistance to radiotherapy as a major obstacle occurs in 80% of osteosarcoma patients (2). Thus, a better understanding of intrinsic radioresistance and the identification a novel radiosensitizer is necessary to improve treatment benefit of osteosarcoma.

Pleckstrin homology-like domain family A member 2 (*PHLDA2*, also known as TSSC3) was the first apoptosis-related gene, paternally imprinted in placenta and most fetal tissues (3). It is a PH domain-containing protein with phosphoinositide-binding capacity, located in the tumor suppressor region p15.5 of human chromosome 11 (4). Our previous study demonstrated that PHLDA2 was associated with the malignant transformation of human osteoblast cells hFOB1.19 via high-throughput microarrays of imprinted genes (5). The downregulation of PHLDA2 has been identified in several human cancers, including osteosarcoma (6, 7). Additionally, the PHLDA2 expression has been considered as an independent prognostic factor with longer survival in osteosarcoma patients, and the inhibition of PHLDA2 has functioned to promote malignant phenotypes of osteosarcoma cells, including proliferation, self-renewal, and therapeutic resistance (8, 9). However, the precise mechanism by which PHLDA2 is suppressed remains poorly understood in osteosarcoma.

MicroRNAs (miRNAs) are a class of small non-coding RNAs of ~22 nucleotides in length and act as regulators of gene expression. miRNAs directly target messenger RNA (mRNA) via a binding complementary sequence in their 3′-untranslated regions (3′-UTR), resulting in mRNA degradation, or translational repression (10). miRNAs have emerged as key components of carcinogenesis, and function as either oncogenes or tumor suppressor genes in the pathogenesis and prognosis of human cancers (11, 12). Accumulating evidence supports a role for miRNA in the modulation of radiosensitivity of tumor cells by affecting DNA damage repair, cell cycle checkpoint, apoptosis, and signal transduction pathways (13). For instance, radiosensitivity was enhanced by the expression of miR-34a and miR-125b via an increased release of apoptosis-induced factors (14, 15), while miR-21 and miR-106 had a radioprotective effect by direct inhibition of pro-apoptotic proteins (16, 17). Furthermore, it has been reported that the deletion of dicer protein led to PHLDA2 overexpression, indicating a regulatory function of miRNAs (18). However, the specific miRNAs that mediate the PHLDA2 inhibition needs to be explored to further see how they affect radiotherapeutic response of osteosarcoma.

For this purpose, a panel of miRNAs that contained potential binding sites of PHLDA2 were detected, and we show that miR-214 is the only miRNA that decreased PHLDA2 expression in osteosarcoma. High levels of miR-214 expression was observed in the majority of osteosarcoma samples, positively associated with lung metastasis. In addition, we further elucidate that miR-214 reduced apoptosis and promoted radioresistance in osteosarcoma via the PHLDA2 inhibition and Akt activation, which is partially antagonized by the expression of PHLDA2 cDNA lacking 3-UTR or PI3K inhibitor treatment. Finally, these findings strengthen a paradigm that miR-214 participates in the radioresistance of osteosarcoma by regulating the PHLDA2/Akt pathway and propose that PHLDA2/Akt could be a novel radiotherapeutic targets in osteosarcoma.

## Materials and Methods

### Cell Culture

The human embryonic kidney cell line HEK293 and human osteosarcoma cell lines MG63, U2OS, HOS, and SaOS2 were purchased from American Type Culture Collections (ATCC, Manassas, USA). SaOS2 was cultured in Mccoy's 5A medium with 500 units/L penicillin, 500 μg/L streptomycin, and 15% fetal bovine serum (Sigma, USA) at 37°C in 5% CO_2_. The other cell lines were maintained as described previously (8).

### Patients and Tissue Samples

All tumor tissues and paired non-tumor tissues were obtained from 30 patients who underwent surgical biopsy for osteosarcoma between 2011 and 2015 at Kunming General Hospital (Yunnan, China), and confirmed by pathologic examination. The patients were limited to the American Joint Committee tumor-node-metastasis system clinical stages T1 to T3, N0/1, M0. None of the patients received any anticancer therapy before sample collection. The samples were immediately frozen in liquid nitrogen for further analysis. This study was carried out in accordance with the recommendations of international guidelines and ethical standards with written informed consent from all subjects. All subjects gave written informed consent in accordance with the Declaration of Helsinki. The protocol was approved by the Institutional Ethics Committee of Kunming General Hospital, China.

### miRNAs, Plasmids, Transfection, and Irradiation

To predict miRNAs with potential binding sites in the 3′UTR of PHLDA2, Targetscan (http://www.targetscan.org/), miRanda (http://www.microrna.org/), and Diana database (http://diana.cslab,ece.nura.gr/) algorithms were used. Mature hsa-miR-214, -134, -193a-3p, -376c, -425, or -499-5p mimics (double-stranded oligonucleotides designed to mimic as endogenous miRNA), anti-hsa-miR-214, or antagomir-214 (antisense RNA oligonucleotides to inhibit the function of miRNA), and individual scrambles (negative control) were purchased from RuiBoBio (Guangzhou, China). A final maximum effective concentration of mimics and inhibitor were 50 nM. The *PHLDA2* CDS sequence without the 3′UTR was cloned into the pcDNA-3.0 plasmid by PCR amplification to construct the pcDNA-PHLDA2 vector as presented previously (8). Transfections were carried out with Lipofectamine 2000 (Invitrogen, USA). External beam radiation was delivered on a 300 KV X ray machine (HITACHI, Japan) at room temperature. Cells were pretreated with PI3K inhibitor LY294002 (Sigma, USA) for 1 h before irradiation.

### Target *in vitro* Reporter Assay

The pmirGLO luciferase reporter vector (Promega, USA) was used for the luciferase assays. The *PHLDA2*-3′UTR was amplified by PCR and inserted into the *Sac*I and *Xba*I sites to construct pmirGLO-wt/*PHLDA2*-3′UTR. Primer sequences were as follows:

*PHLDA2*-3′UTR-F 5′-AGCC*GAGCTC*GCCCGCCGCGGGCCATACGCTG-3′ *PHLDA2*-3′UTR-R 5′-GC*TCTAGA*GCGCAGATGACACGATTCATTTATTC-3′.

The potential miR-214-binding sites in *PHLDA2*-3′UTR were mutated using Quikchange Mutagenesis Kit (Agilent Technologies, USA) to develop the pmirGLO-mut/*PHLDA2*-3′UTR vector. Cells were seeded in 12-well-plates at a density of 1 × 10^5^ cells/well, and co-transfected with miR-214 mimics or scrambles and pmirGLO-wt/*PHLDA2*-3′UTR or pmirGLO-mut/*PHLDA2*-3′UTR vector per well for 48 h by using the dual luciferase reporter assay system (Promega, USA). The empty pmirGLO reporter vector was transfected as control. The mean value of relative luciferase activities from the cells transfected with the empty vector and scrambles was set as 1.0.

### Quantitative Real-Time Reverse Transcriptase PCR Analysis

Total RNA from cell lines and tissue samples was isolated using TRIZOL reagent (Invitrogen, USA). Complementary DNA (cDNA) was synthesized from 10 ng of RNA using the Taqman miRNA Reverse Transcription Kit with specific stem-loop reverse transcription primers (Applied Biosystems, USA). A quantitative PCR reaction was performed using TaqMan Universal PCR master mix with miRNA specific PCR-primers (Applied Biosystems, USA) on the ABI Prism 7,900 (Applied Biosystems, USA). The relative expression of expression was analyzed using the 2^−ΔΔCt^ method after normalization with the value of U6 small nuclear RNA (19).

### Western Blot Analysis

Total protein was extracted using a protein extraction reagent (Roche, Switzerland), and measured with a BCA protein assay kit (Pierce Biotechnology, USA). Equal amounts of the protein (40 μg) were separated by 12–15% SDS-polyacrylamide gels and transferred onto nitrocellulose membranes. Membranes were blocked with 5% fat-free milk in TBST (Tris-buffered saline containing 0.1% Tween 20) at room temperature for 1 h, and subsequently incubated with the primary antibody at 4**°**C overnight. Anti-Akt, anti-phospho-Akt, anti-cleaved caspase-3, anti-cleaved caspase-9, and anti-cleaved PARP antibody were purchased from Cell Signaling Technology (1:1,000); Anti-PHLDA2 antibody was obtained from Abcam (1:1,000); GAPDH antibody as an internal control was obtained from Santa Cruz Technology (1:10,000). After incubation with goat anti-mouse or anti-rabbit IgG at 37**°**C for 1 h, all bands were detected using the ECL chemiluminescence kit (Pierce Biotechnology, USA).

### Colony Formation Assay

Cells were plated into six-well-plates with the appropriate number of cells (1,000, 2,000, 4,000, 8,000, or 16,000 cells/well, serially), and each dilution was irradiated with single doses of 0, 2, 4, 6, or 8 Gy after 24 h. Fourteen days later, cells were fixed with acetone and stained with crystal violet. Colonies with equal or >50 cells were counted. Plating efficiency (PE) was measured for each cell line, and Surviving fractions were calculated using the equation SF = (number of colonies formed)/(number of cell seeded × plating efficiency for sham irradiated group) × 100%, as described previously (20). The value of the sensitization enhancement ratio (SER10) were used to evaluate the survival curve.

### Apoptosis Assay

Apoptosis was determined by Annexin V–fluorescein isothiocyanate (FITC) and propidium iodide (PI) staining according to the protocol (BD Bioscience, USA). Briefly, cells were harvested gently and washed twice with cold PBS, then 1 × 10^6^ cells/ml were resuspended in a 100 μl binding buffer containing 5 μl FITC-labeled Annexin V and 5 μl PI. After incubation for 15 min at room temperature, the samples were analyzed on a FACS Calibur (Becton-Dickinson, CA). Unstained cells were classified as “live,” those stained by Annexin V only were classified as “early apoptotic,” those stained by both Annexin V and PI were classified as “later apoptotic,” and those stained by PI only were classified as “dead” cells.

### Xenografts in Nude Mice

U2OS cells transfected with antagomiR-214 or scrambled control (2 × 10^6^ in 100 μl PBS/mouse) were subcutaneously injected into the right fore legs of 4-week-old nude mice. Eight days later, a mean diameter of 6–8 mm xenografts was seen in mice, and mice were randomly divided into 4 groups (5 mice/group), including scrambles, antagomiR-214, scrambles plus IR, and antagomiR-214 plus IR. 10 Gy fractionated radiation (5 Gy/Fx separated by a 48-h interval) was delivered to local tumor. The tumor volumes were calculated every 2 days using the formula = (width^2^ × length)/2, and Mice were sacrificed 4 weeks after irradiation treatment. Efforts were made to ensure the animals suffered minimally. All animal experiments were approved by the Animal Care Committee of Kunming General Hospital, China.

### Histology and Immunohistochemistry Assays

The xenograft tumors were fixed in 10% formalin, dehydrated in a graded series of alcohol, and embedded in paraffin. Four micrometer thick slices were stained with hematoxylin and eosin, and immunohistochemistry (IHC) was subsequently performed as previously described (21). Briefly, the slices were incubated with the primary antibody against PHLDA2 (Abcam, USA), Ki67 and cleaved caspase-3 (Santa Cruz biotechnology, CA) at 4°C overnight, and subsequently stained with a biotinylated secondary antibody (Zhongshan, China) at room temperature for 1 h. Negative controls without a primary antibody were processed in parallel.

### Statistical Analysis

Data were presented as the means ± standard deviation (SD). Two-tailed student's *t*-tests were used to evaluate differences in values between two experimental groups. The Pearson correlation test was used to analyze the correlation between the miR-214 and PHLDA2 expression in tissue samples. The Fisher's exact test was used to analyze the relationship between miR-214 expression and each clinicopathologic parameter. A *P* < 0.05 was considered to be statistically significant between groups.

## Results

### miR-214 Downregulates PHLDA2 Protein by Targeting its 3′-UTR and Is Differentially Expressed in Osteosarcoma Cell Lines

**Figure 1:**
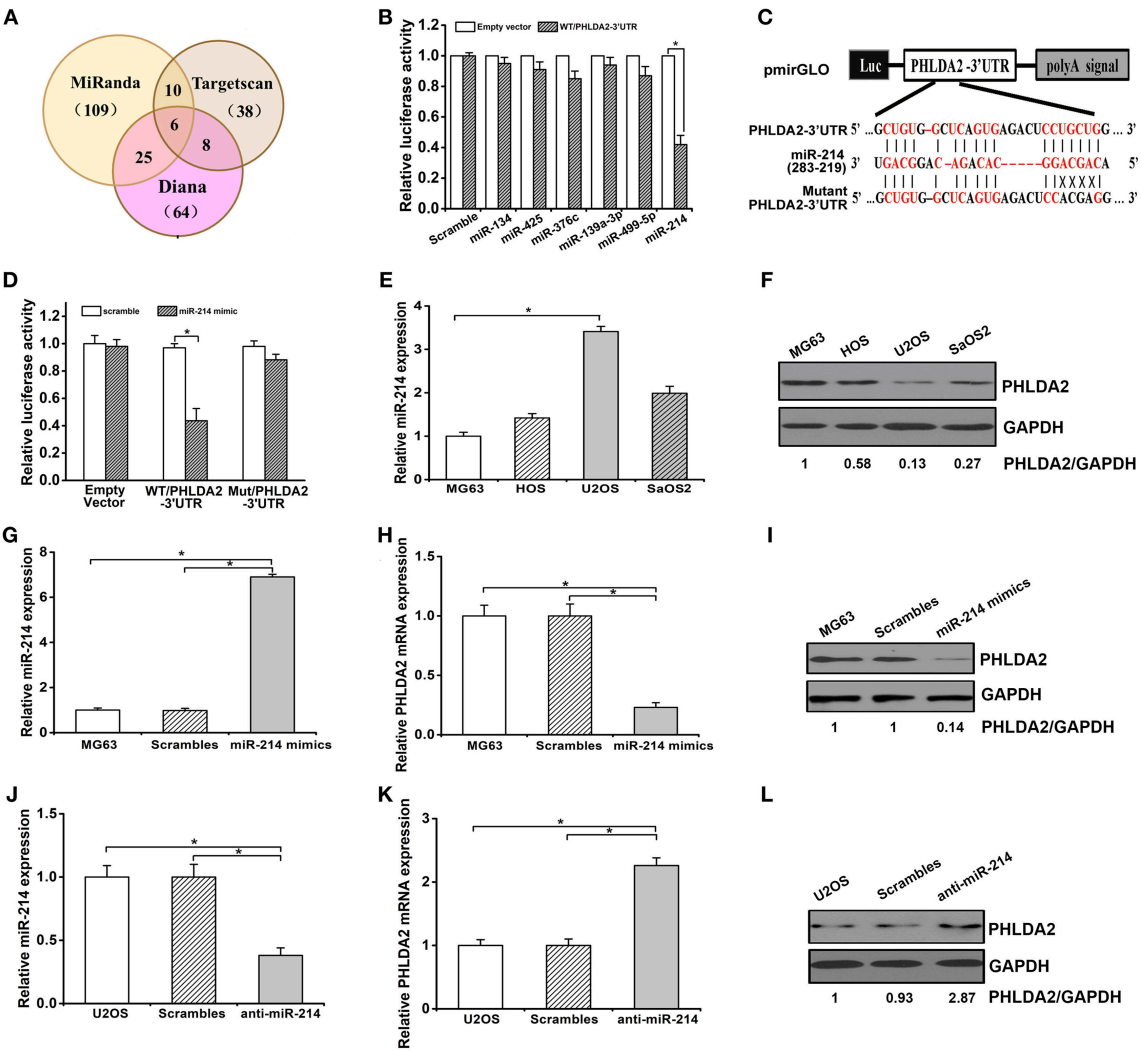
miR-214 targets PHLDA2. **(A)** Bioinformatics analysis of candidate miRNAs with potential binding sites in PHLDA2 3′UTR. Digits in brackets represented the total number of miRNAs predicted by each database. **(B)** Luciferase assay showed miR-214 mimics inhibited reporter activity with wild-type PHLDA2-3′UTR in HEK293T cells. **(C)** Schematic illustration of a pmirGLO luciferase vectors with PHLDA2 3′-UTR and sequence alignment of *PHLDA2*-3′UTR (*top*) with hsa-miR-214 (*middle*). *Bottom*, substitution of four bases (UGCU to ACGA) at the binding sites of miR-214 to construct the mutate vector (Matant *PHLDA2* 3′UTR). **(D)** The inhibition of reporter activity was abolished by mutant vector with *PHLDA2*-3′UTR. **(E,F)** qRT-PCR and western blot for miR-214 expression and PHLDA2 protein in four osteosarcoma cell lines, respectively. **(G)** qRT-PCR for miR-214 expression in MG63 cells without and with scrambles or miR-214 mimics. **(H,I)** miR-214 mimics decreased the mRNA and protein level of PHLDA2 in MG-63 cells, respectively. **(J)** qRT-PCR for miR-214 expression in U2OS cells without and with scrambles or anti-miR-214. **(K,L)** Anti-miR-214 upregulated the mRNA and protein level of PHLDA2 in U2OS cells, respectively. Data are presented as mean ± SD of three independent experiments. ^*^P < 0.05.

To determine whether miRNAs could regulate *PHLDA2*, we searched miRNAs containing seed sequence to match 3′-UTR of PHLDA2 via Targetscan, miRanda, and Diana database. Predicted candidates in the overlap of the above databases were identified as miR-134, miR-193a-3p, miR-376c, miR-425, miR-214, and miR-499-5p (Figure 1A). After cotransfection of individual miRNA mimics and pmirGLO vector with wild-type *PHLDA2*-3′UTR in HEK293 cells, a dramatic inhibition of reporter gene activity was only observed by miR-214 mimics (Figure 1B). In contrast, this effect was abolished by mutant miR-214-targeting sequences in the PHLDA2-3′-UTR, and only an 8% reduction of luciferase activity was observed, suggesting that the mutated sites were critical to miR-214 binding (Figures 1C,D). We then measured the endogenous expression of miR-214 by qPCR in four osteosarcoma cell lines, MG63, U2OS, SaOS2, and HOS. We found that the miR-214 expression in U2OS cells was significantly higher than that in others, with a low level of PHLDA2 protein, while MG63 cells with the lowest miR-214 expression showed an 87% increase of PHLDA2 protein than U2OS cells (Figures 1E,F). To further test whether miR-214 could alter the expression of PHLDA2 in osteosarcoma, miR-214 mimics, and anti-miR-214 were transfected into MG-63 and U2OS cell lines, respectively. The overexpression of miR-214 was confirmed after transfecting miR-214 mimics into MG63 cells (Figure 1G), and miR-214 mimics showed a significant decrease of PHLDA2 mRNA and protein levels compared to a scrambled control transfection (Figures 1H,I). The miR-214 expression was obviously reduced via anti-miR-214 transfection in U2OS cells (Figure 1J), associated with a >2-fold increase of both mRNA and protein level of PHLDA2 in comparison with scrambled control (Figures 1K,L).

### Frequent Upregulation of miR-214 Correlates Significantly With PHLDA2 Low Expression and Lung Metastasis in Osteosarcoma Tissues

**Figure 2:**
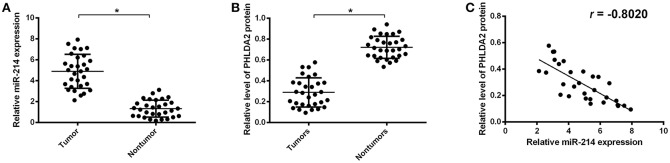
Expression of miR-214 and PHLDA2 in osteosarcoma tissues. **(A)** qRT-PCR analysis of miR-214 expression in paired tumor and non-tumor specimens, *n* = 30. **(B)** Expression of PHLDA2 protein in paired tumor and non-tumor specimens by western blot, *n* = 30. **(C)** Regression analysis for the correlation of miR-214 and PHLDA2 protein in tumor specimens. Data are presented as mean ± SD of three independent experiments. ^*^P < 0.05.

To further validate the potential association of miR-214 and PHLDA2, their expressions were analyzed in 30 osteosarcoma samples. The qPCR assay showed that miR-214 levels were up-regulated in tumor specimens with the median expression of 4.95, which was significantly higher than that of paired non-tumor tissues (Figure 2A). Interestingly, a significantly lower expression of PHLDA2 was observed in tumor samples with a mean value of 0.29 compared to paired normal tissue (Figure 2B). In the high miR-214 subgroup, 12 tumor samples showed weaker or a lack of PHLDA2 expression (12/16), and significant negative relativity with miR-214 and PHLDA2 was observed (Figure 2C, *r* = −0.802). As shown in Table 1, miR-214 expression is not correlated with gender, age, histology, differentiation, and location of primary tumor, but an increased incidence of lung metastasis in the high miR-214 subgroup was observed during the follow-up period (*P* = 0.026).

**Table 1:** Correlation between miR-214 expression and clinicopathologic features of patients with osteosarcoma.

**Parameters**	**Total**	**No. of patients^†^**		**Fisher exact test**
		**High miR-214**	**Low miR-214**	***P*-value**
**Gender**				0.442
Male	20	12	8	
Female	10	4	6	
**Age**				0.713
≤ 16.5	17	10	7	
>16.5	13	6	7	
**Histology**				0.769
Osteoblastic	19	9	10	
Chondroblastic	6	4	2	
Fibroblastic	5	3	2	
**Differentiation**				0.292
Well differentiation	5	4	1	
Moderately differentiation	12	4	8	
Poorly differentiation	11	7	4	
Other	2	1	1	
**Location of Primary Tumor**				
Femur	16	10	6	0.494
Tibia	11	5	6	
Other	3	1	2	
**Lung metastasis**				0.026^*^
Present	16	12	4	
Absent	14	4	10	

* *P < 0.05 vs. lung metastasis absent*.

† *The median expression of miR-214 in osteosarcoma tissues was used as a cutoff to divide samples into high or low subgroup*.

### miR-214 Knockdown Confers Radiosensitivity of Osteosarcoma *in vitro* and *in vivo*

**Figure 3:**
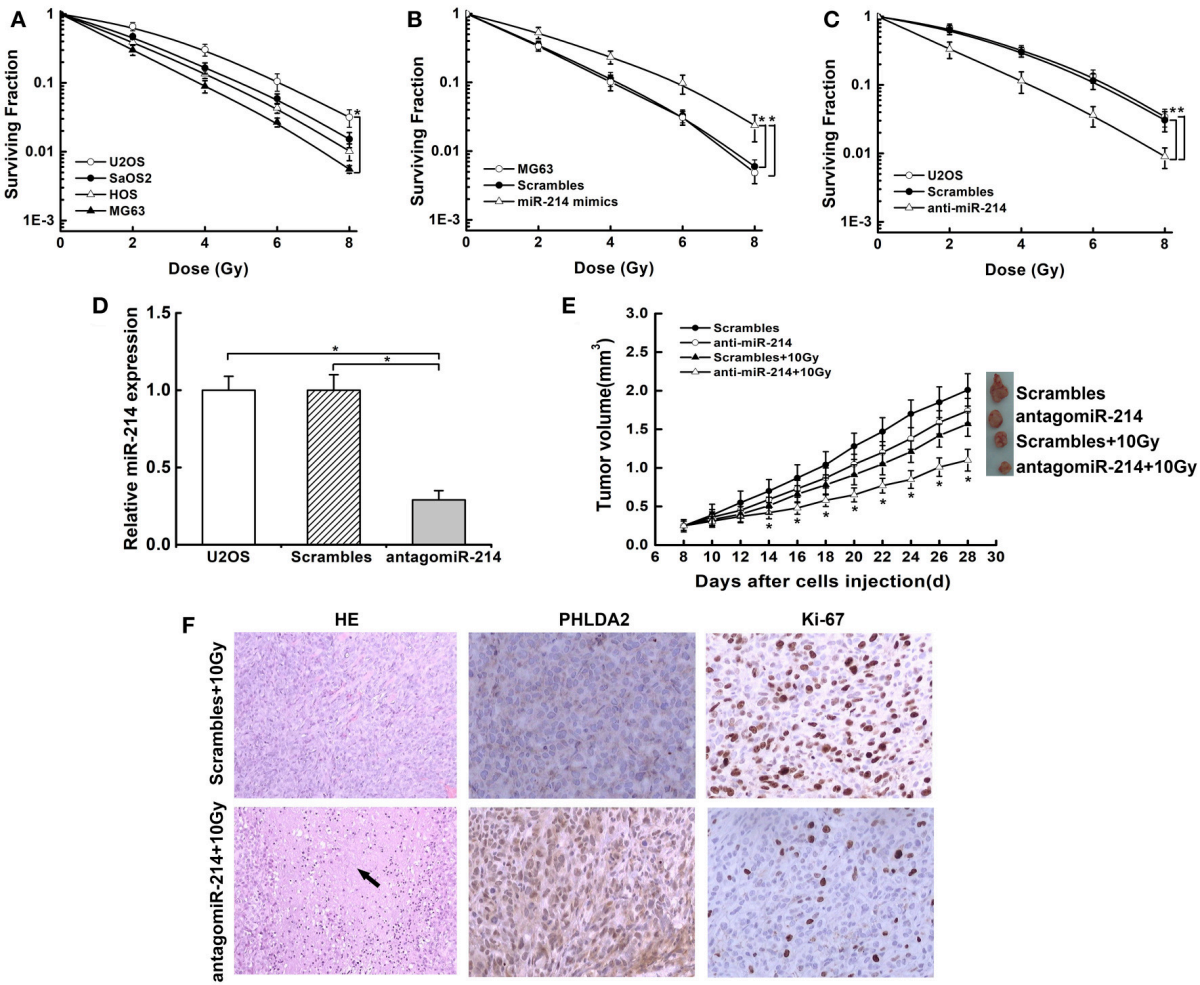
Effects of miR-214 on radiosensitivity of osteosarcoma *in vitro* and *in vivo*. **(A)** Clonogenic survival assays were performed in four osteosarcoma cell lines. An upward and rightward shift of survival curve indicated resistance to irradiation. **(B)** Treatment of miR-214 mimics decreased radiosensitivity of MG63 cells. **(C)** Treatment of anti-miR-214 increased sensitivity of U2OS cells to irradiation. **(D)** AntagomiR-214 decreased miR-214 expression in U2OS cells via qRT-PCR assay. **(E)** miR-214 depletion significantly inhibited the xenograft growth after combined treatment with irradiation, and representative photographs of xenografts from each group were shown. **(F)** Representative images of H&E staining and IHC analysis of PHLDA2 and Ki67 in xenograft tumors from miR-214 depleted-U2OS cells with irradiation (magnification: 100x for H&E staining, 200x for IHC). Black arrow represented necrosis. Data are presented as mean ± SD of three independent experiments. ^*^P < 0.05.

To investigate whether miR-214 played a role in radio-response, we examined the probable association of endogenous miR-214 with radiosensitivity in osteosarcoma cells by colony survival assay. We found that the capacity of colonies was obviously diverse among the four cells. MG63 cells with a low miR-214 expression showed sensitivity to radiation, while U2OS cells with a high miR-214 level were more radioresistant than others (Figure 3A). We further verified the above effect of miR-214 by modulating its expression and found that miR-214 overexpression boosted the capacity of colony formation, and it led to a 22% decreased SER10 value of radiosurvival curve compared with scramble-transfected MG63 cells (SER10: 0.78 vs. 1, *P* < 0.05, Figure 3B). In contrast, colony formation efficiency reduced significantly upon miR-214 depletion, with a 45% increase of SER10 value than scramble-transfected U2OS cells (SER10:1.45 vs. 1, *P* < 0.05, Figure 3C). In addition, this radiosensitive effect of miR-214 depletion was also examined through an *in vivo* mouse model. The tumor incidence was 100% in all groups. We measured the volume of xenografts every two days and found that xenografts formed by miR-214 depleted-U2OS cells grew slowly compared with scrambled group without IR. Importantly, a more significant growth inhibition was observed when miR-214 depletion was combined with 10 Gy irradiation (Figures 3D,E), with more necrosis compared with scrambles by HE staining. Additionally, an increased level of PHLDA2 was observed in combination with miR-214 depletion and IR, along with a reduction of the Ki67 proliferation index using immunohistochemistry assays (Figure 3F). Our findings suggest that miR-214 depletion could antagonize the radioresistance of osteosarcoma *in vitro* and *in vivo*.

### miR-214 Knockdown Enhances IR-Induced Apoptosis of Osteosarcoma Cell Lines

**Figure 4:**
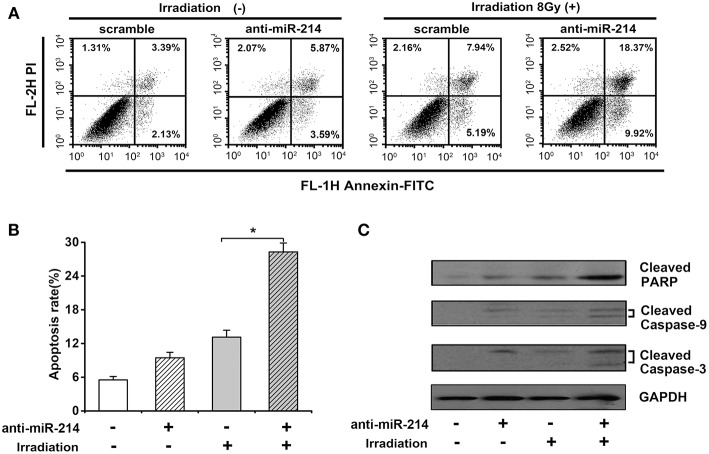
Effects of miR-214 on irradiation-induced apoptosis in osteosarcoma. **(A)** Apoptotic analysis in scramble- or anti-miR-214-transfected U2OS cells with and without irradiation. Cells in the right quadrant represent early *(lower right)* and late *(upper right)* apoptotic cells, respectively. **(B)** Quantification of apoptotic ratio of the indicated groups. **(C)** Western blot analysis for cleaved caspase-3, cleaved caspase-9, cleaved PARP, and GAPDH (for protein normalization) was performed in scramble- or anti-miR-214-transfected U2OS cells without and with irradiation. Data are presented as mean ± SD of three independent experiments. ^*^P < 0.05.

Given the importance of apoptosis in radiosensitivity, the effect of miR-214 depletion on radiation-induced apoptosis was analyzed by annexin V staining and necrotic cells analyzed by propidium iodide (PI) staining. As shown in Figures 4A,B, a minor increase of apoptotic percentage was observed in anti-miR-214-transfected U2OS cells compared to scrambled cells without IR treatment. However, miR-214 depletion significantly enhanced IR-induced apoptosis, with a 15.16% increase of apoptotic ratio compared with scrambles in response to IR (Figures 4A,B). To further confirm the above effect of miR-214 depletion and IR, several apoptotic markers were analyzed. Western blot assay showed that caspase-dependent apoptotic pathway was activated in anti-miR-214-transfected U2OS cells in the presence of IR, with an obvious increase of cleaved caspase-9 and caspase-3 compared with those in combined treatment of scrambles and IR. Moreover, a similar trend in cleaved PARP and PHLDA2 protein was also found in miR-214 depletion and IR treatment (Figure 4C). These data suggest that miR-214 depletion may confer sensitivity to radiation-induced apoptosis.

### PHLDA2/AKT Signaling Pathway Mediates miR-214-Induced Radioresistance

**Figure 5:**
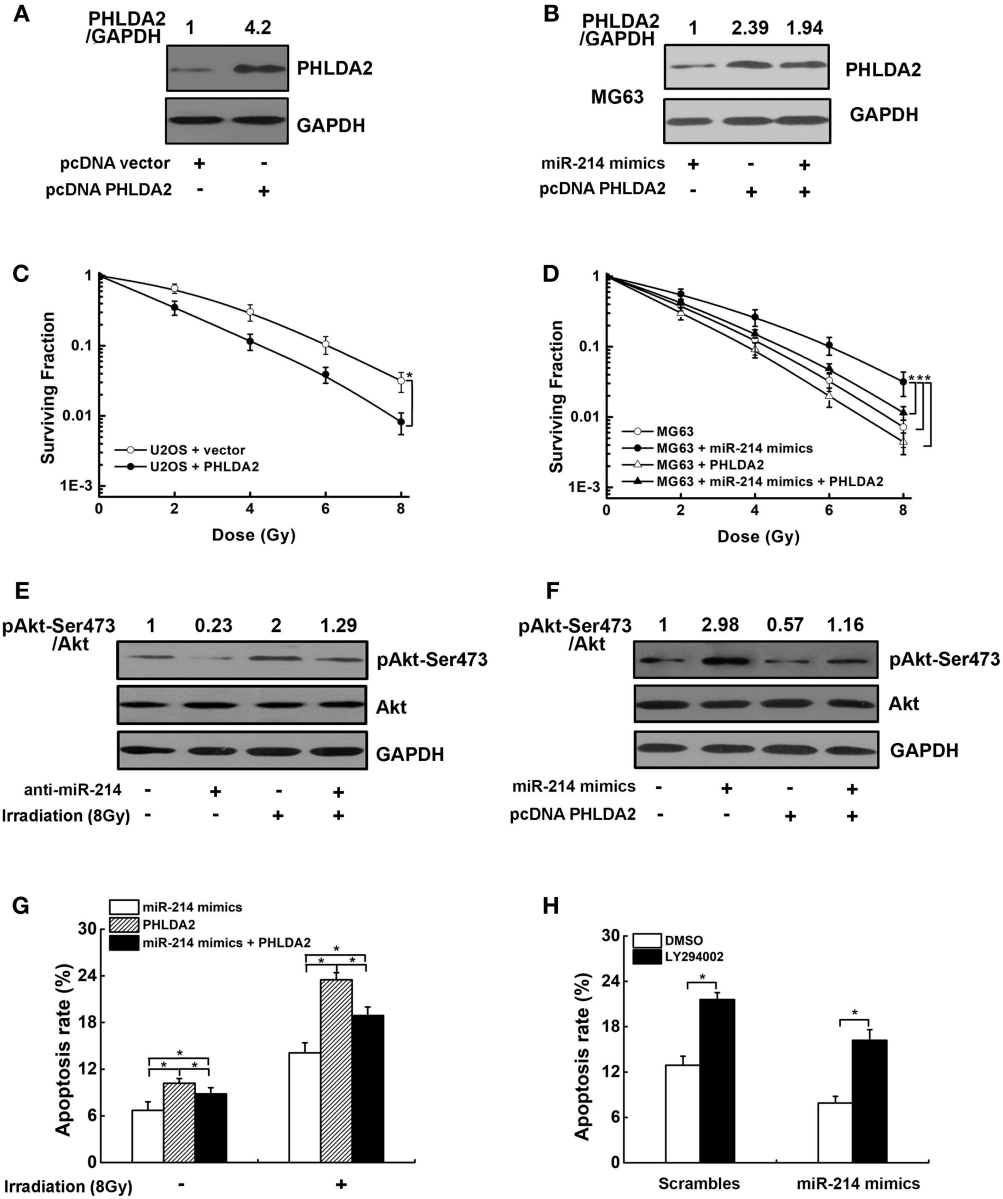
Effects of miR-214 on the PI3K/Akt pathway. **(A,C)** The expression of PHLDA2 protein in U2OS cells after reintroduction of *PHLDA2*-expressing vector lacking 3′UTR (pcDNA PHLDA2). Overexpression of PHLDA2 significantly increased the radiosensitivity. **(B,D)** PHLDA2 vector and miR-214 mimics was co-transfected into MG63 cells. The overexpression of PHLDA2 was confirmed by western blot, which significantly antagonized the miR-214-induced radioresistance. **(E)** Western blot analysis for pAkt-Ser473, Akt, and GAPDH (for normalization) in miR-214-depleted U2OS cells without and with irradiation. **(F)** Western blot analysis of pAkt-Ser473, Akt and GAPDH (for normalization) in MG63 cells. The activation of miR-214 on Akt phosphorylation was dramatically abolished by PHLDA2 vector. **(G,H)** Overexpression of PHLDA2 and treatment with a PIK3CA inhibitor LY294002 significantly abrogated the inhibitory effect of miR-214 on IR-induced apoptosis. Data are presented as mean ± SD of three independent experiments. ^*^P < 0.05.

To explore whether the radioresistant effect of miR-214 was associated with its target PHLDA2, a *PHLDA2*-expressing vector lacking 3′UTR was transfected into U2OS cells, which could escape miR-214 regulation and attenuate its function. The ectopic expression of PHLDA2 protein was confirmed by western blot (Figure 5A). We found that the PHLDA2 overexpression significantly enhanced the radiosensitivity of U2OS cells, with a lower capacity of colony formation compared to empty vector-transfected U2OS cells (Figure 5C). Furthermore, this PHLDA2 vector was co-transfected into MG63 cells with miR-214 mimics, and we found that the ectopic expression of PHLDA2 significantly antagonized the miR-214-induced radioresistance, with a 16% increase of SER10 value compared with miR-214-expressing cells (SER10: 0.92 vs. 0.79, *P* < 0.05, Figures 5B,D).

We next investigated the molecular mechanism by which miR-214-triggered PHLDA2 downregulation led to radiation resistance in osteosarcoma cells. Because previous studies showed the PHLDA2 expression could regulate Akt activation (22), we suspected that the above effect of miR-214 could be due to PI3K/Akt pathway. To test this, phosphorylated Akt (p-Akt-Ser473) was measured in scramble- and anti-miR-214-transfected U2OS cells with IR. As shown in Figure 5E, the level of phosphorylated Akt was obviously reduced in miR-214-depleted cells compared to scrambled control without IR treatment. We further observed the miR-214 depletion could remarkably antagonize IR-induced Akt activation (Figure 5E). In contrast, a 2.9-fold increase of phosphorylated Akt protein was observed in miR-214 expressing-MG63 cells compared to scrambled control. Moreover, the activation of miR-214 on Akt phosphorylation was dramatically abolished by PHLDA2 overexpression (Figure 5F). In addition, apoptosis assay showed that ectopic expression of PHLDA2 in miR-214-expressing MG63 cells significantly abrogated the inhibitory effects of miR-214 on IR-induced apoptosis, and the same trend was also observed in miR-214-expressing MG63 cells after treatment with a PIK3CA inhibitor LY294002 (Figures 5G,H).

## Discussion

Our analysis revealed that miR-214 was a negative regulator of PHLDA2, which is overexpressed in osteosarcoma tissues and negatively associated with lung metastasis. We also confirmed that miR-214 expression is required for maintaining radioresistance to apoptosis in osteosarcoma, which is mediated by the repression of PHLDA2 and activation of Akt pathway.

The potential tumor suppressor role of PHLDA2 with downregulated protein expression has been explored in several human cancers. The involvement of RanBP9 in regulating the PHLDA2 expression has been described previously (23); however, the transcriptional mechanism by which PHLDA2 is repressed in osteosarcoma has been elusive. Here, we provide evidence that miRNA can be directly responsible for PHLDA2 repression. In this study, we use bioinformatics analysis to predict a set of miRNAs with potential binding sites for PHLDA2 3′-UTR and found that miR-214 negatively regulated the PHLDA2 expression. It is possible that interaction between miRNAs and mRNA is tissue- and cell-specificity, which also depends on the sequence specificity, such as AU-rich nucleotide composition and relative position of binding sites to stop codon (24, 25).

miR-214 has been previously found to be involved in osteoblast differentiation and osteoblastic bone formation in skeletal disorders (26). Accumulating studies have reported that the dysregulation of miR-214 is causative for carcinogenesis, which may function as either an oncogene or a tumor suppressor gene dependent on specific types of tumor. Overexpression of miR-214 was observed in gastric, melanoma and pancreatic tumors (27, 28). We show here that miR-214 expression is higher in osteosarcoma samples than in adjacent non-tumor tissues, which is in accordance with previous reports (29). In addition, we first found that the upregulation of endogenous miR-214 is related to lung metastasis in osteosarcoma patients. Although miR-214 has previously been described as a poor prognostic marker in pediatric osteosarcoma, no significant association between them is identified in our study. It is likely that a small sample size and varied therapeutic treatments have an effect on the association. A longer follow-up and increasing sample size could help in dissecting the role of miR-214 expression as a suitable biomarker for osteosarcoma.

miR-214 has been known as a major driver of chemoresistance in several cancers (30–32). Furthermore, we provide clear evidence that miR-214 is directly involved in the radiotherapeutic response of osteosarcoma. In this study, we show that the miR-214 overexpression facilitates radioresistance in osteosarcoma cells. On the contrary, the radiosensitivity is observed upon combined miR-214 depletion and treatment with IR. In this case, the IR-induced apoptosis was enhanced along with an increased activity of caspase-9 and caspase-3 in miR-214 depleted cells. The intrinsic apoptosis with mitochondrial release of cytochrome c is known to be a major pathway stimulated by irradiation, with the formation of apoptosome complex containing cytochrome c/APAF1/caspase-9. The initiator caspase-9 activating the effector caspase-3 subsequently leads to apoptosis via the post-mitochondrial-mediated caspase cascade (33, 34). Moreover, PARP-1 is one of several known cellular substrates of caspases, and cleavage of PARP-1 by caspases has been considered to be a hallmark of apoptosis (35). Here, we also show the high level of cleaved PARP via the concurrent treatment with miR-214 and IR. Our findings suggest that synergistic pro-apoptosis via miR-214 depletion with IR may contribute to alleviate the radioresistance of osteosarcoma.

What molecular mechanism, exactly, is responsible for miR-214-rendered radioresistance in osteosarcoma? We examined the PI3K/Akt pathway, as its role in triggering a cascade of multiple signals that regulate survival, metastasis, and therapeutic resistance has been demonstrated previously (36). We show that the miR-214 overexpression increased the activation of phosphorylated Akt, and miR-214 depletion contrarily reduced the phosphorylation of Akt, which exhibited a similar tendency with the previous literature (37). Further mechanistic analysis finds that the introduction of PHLDA2 cDNA lacking the 3′-UTR partially abrogates miR-214-induced Akt activation, providing evidence that PHLDA2 is directly involved in the miR-214-mediated inhibition of PI3K/Akt signaling. PHLDA2 is known as a pleckstrin homology domain (PH domain) only protein as its homolog PHLDA3. It is important to note that PHLDA3 functions as a unique Akt inhibitor through the depletion of membrane-bound PIPs (38). Given the importance of PH domain in PIP3-binding ability, it is possible that PHLDA2 may repress Akt activation by interfering with Akt translocation to the plasma membrane via direct competition with Akt to PIPs (39). A recent report showed also that PHLDA2 inhibited Akt activity via forming a ternary complex with RanBP9 and Src, which facilitated mitochondrial-associated anoikis (23). Furthermore, we observed that PHLDA2 overexpression antagonizes radioresistance in miR-214-upregulated cells in part as a consequence of increased apoptosis upon radiation treatment. Thus, we propose that the radioresistance mediated by miR-214 is ascribed to recruitment of its downstream target PHLDA2 to PI3K/Akt pathway. Since the “one hit-multiple targets” strategy has been considered as a valuable approach to establish a general paradigm of radiosensitive mechanism, the identification of novel miR-214-targeted genes not only could help us better understand the radiosensitive network, but this information could help us devise improved therapies for osteosarcoma.

## Conclusions

In summary, our results reveal that upregulation of miR-214 is a frequent event and negatively associated with PHLDA2 expression in osteosarcoma. Furthermore, miR-214 depletion enhanced apoptosis and radiosensitivity by inhibiting the Akt pathway via PHLDA2. The miR-214/PHLDA2/Akt axis provides a new avenue toward understanding the mechanism of radiosensitivity and may be a potential target for osteosarcoma intervention.

## Ethics Statement

This study was carried out in accordance with the recommendations of international guidelines and ethical standards with written informed consent from all subjects. All subjects gave written informed consent in accordance with the Declaration of Helsinki. The protocol was approved by the Institutional Ethics Committee of 920th Hospital of Joint Logistics Support Force, China. All animal experiments were approved by the Animal Care Committee of 920th Hospital of Joint Logistics Support Force, China.

## Author Contributions

YL, XS, and HC conceived and designed the experiments. YL, YM, WM, MH, and BS performed the experiments. YL, ZL, QL, and YW analyzed the data. YL wrote the paper. HC, XS, and YL revised the paper.

### Conflict of Interest Statement

The authors declare that the research was conducted in the absence of any commercial or financial relationships that could be construed as a potential conflict of interest.
